# Genomic Characterization of a Carbapenem-Resistant *Acinetobacter pittii* Strain Harboring Chromosome-Borne *bla*_NDM-1_ from China

**DOI:** 10.3390/pathogens14101037

**Published:** 2025-10-13

**Authors:** Wenjuan Liu, Haixia Wang, Weijian Zhu, Xiaobin Li, Ying He, Wen Su

**Affiliations:** 1 Department of Pulmonary and Critical Care Medicine, Yantaishan Hospital, Yantai 264000, China; 15684123265@163.com (W.L.); haixia998877@163.com (H.W.); 2 Department of Hematology & Rheumatology, Zhuhai People’s Hospital (The Affiliated Hospital of Beijing Institute of Technology, Zhuhai Clinical Medical College of Jinan University), Zhuhai 519000, China; jjian2003@126.com; 3 Department of Pulmonary and Critical Care Medicine, Zhuhai People’s Hospital (The Affiliated Hospital of Beijing Institute of Technology, Zhuhai Clinical Medical College of Jinan University), Zhuhai 519000, China; 4 Guangdong Provincial Key Laboratory of Tumor Interventional Diagnosis and Treatment, Zhuhai People’s Hospital (The Affiliated Hospital of Beijing Institute of Technology, Zhuhai Clinical Medical College of Jinan University), Zhuhai 519000, China; 5 Zhuhai Precision Medical Center, Zhuhai People’s Hospital (The Affiliated Hospital of Beijing Institute of Technology, Zhuhai Clinical Medical College of Jinan University), Zhuhai 519000, China

**Keywords:** *Acinetobacter pittii*, chromosome-borne *bla*_NDM-1_, whole-genome sequencing, Tn*125*, plasmid

## Abstract

New Delhi metallo-beta-lactamase (NDM)-producing *Acinetobacter* spp. have been reported worldwide and become a global threat to clinics. This study aimed to characterize the genomic features of the carbapenem-resistant *Acinetobacter pittii* strain AP8900 harboring chromosome-borne *bla*_NDM-1_. The genome of strain AP8900 was fully sequenced using Illumina and PacBio platforms. Genome analyses revealed that the chromosome-borne *bla*_NDM-1_ of strain AP8900 was located on the Tn*125* bracketed by two copies of IS*Aba125* in the same orientation. So far, only five strains of *A. pittii* with complete genomes harboring chromosome-borne *bla*_NDM-1_ were found (four from China and one from the USA), all carrying nearly identical Tn*125* carried by the strain AP8900. Furthermore, the Tn*125* of strain AP8900 in this study was also distributed in other species, mainly *Acinetobacter* spp. Notably, the Tn*125* carried by AP8900 also found in *Proteus mirabilis*, *Klebsiella pneumoniae*, and *Morganella morganii*. In addition, two antibiotic resistance plasmids were found in strain AP8900, and the configuration “*sul2*- *glmM*” was found on both pAP8900-1 (*ISAba1*-*sul2*-*glmM*-IS*Vsa3*-IS*1006*) and pAP8900-2 (∆IS*Aba2*-*sul2*-*glmM*-IS*17*). This study delivers comprehensive insights into the characteristics and diversity of chromosome-borne *bla*_NDM-1_ in *A. pittii*. The complete genome of *A. pittii* AP8900 strain from southern China provides important data for the analysis of antimicrobial resistance in this region.

## 1. Introduction

According to the Ambler Classification system, β-lactamases can be divided into four classes (A, B, C, and D) [1]. New Delhi metallo-beta-lactamase (NDM), an Ambler class B beta-lactamase, can hydrolyze nearly all beta-lactam antibiotics, including carbapenems [2]. The first report of NDM-1 was in a *Klebsiella pneumoniae* isolate recovered in a Swedish patient who had been hospitalized in New Delhi, India, in 2008 [3]. Since then, NDM-1 and its variants have been identified in different species of *Enterobacteriaceae*, *Pseudomonas*, and *Acinetobacter* [4]. According to the records of the beta-lactamase database [5], on 9 January 2025, more than 79 variants of NDM have been identified. The NDM-1 and its variants are continuously spreading globally [4,6], and NDM-producing bacterial pathogens have posed significant therapeutic challenge for clinicians and have attracted considerable attention [7,8].

*Acinetobacter* spp. are frequently associated with nosocomial infections, such as aspiration pneumonia, catheter-associated bacteremia, soft tissue infection, and urinary tract infection [9,10]. Infections caused by *Acinetobacter* spp. are among the most difficult to treat because of the multidrug resistance in this genus mediated by the horizontal acquisition of resistance genes or overexpression of the efflux pumps system [11,12]. Although *Acinetobacter baumannii* receives more attention in genus *Acinetobacter*, *Acinetobacter pittii* is an emerging opportunistic nosocomial pathogen [13,14], which is the important member of the *Acinetobacter calcoaceticus-baumannii* complex [15]. *A. pittii* has been an emerging concern in nosocomial infection because of its increasing prevalence and multidrug resistance [16].

The *bla*_NDM_ genes have appeared in various genetic contexts, implying that multiple mechanisms have facilitated the mobilization of *bla*_NDM_ [17]. The *bla*_NDM_ has been found to be located on many conjugative and mobilizable plasmids in different species of *Enterobacteriaceae* and *Acinetobacter* [4,18,19]. In addition, numerous mobile elements, including IS*Aba125*, IS*26*, IS*3000*, IS*5*, IS*CR1*, Tn*125*, Tn*3*, and Tn*3000*, are thought to play crucial roles in the dissemination of *bla*_NDM_ [4,20]. In *Acinetobacter* spp., the *bla*_NDM_ has been reported to be embedded in composite transposon Tn*125* [21,22,23], bracketed by two copies of the IS*Aba125* in the same orientation, all carrying the nearly identical Tn*125* carried by AP8900, which was initially identified in *A. baumannii* isolate 161/07, *A. baumannii* isolate JH, and *A. baumannii* isolate ML [22]. Current evidence suggests that Tn*125* serves as the primary vehicle for *bla*_NDM_ gene dissemination within *Acinetobacter* populations, with the IS*Aba125* elements likely playing a crucial role in its horizontal transfer mechanisms [22]. Currently, studies specific to the characteristics and diversity of chromosome-borne *bla*_NDM_ in *Acinetobacter* spp. are scarce. As whole-genome sequencing data grows, there is a demand for extensive analysis of chromosome-borne *bla*_NDM_ in *Acinetobacter* spp.

In the present study, we characterized the genomic features of the carbapenem-resistant *A*. *pittii* strain AP8900 harboring chromosome-borne *bla*_NDM-1_. This study provides deep insights into the characteristics and diversity of chromosome-borne *bla*_NDM_ in *Acinetobacter* spp. as well as the acquisition and spread of the *bla*_NDM_.

## 2. Materials and Methods

### 2.1. Isolation and Characterization of the Strain AP8900

The strain AP8900 was isolated from the sputum sample obtained from a 60-year-old patient at the Zhuhai People’s Hospital in year 2022. The fully automatic VITEK 2 COMPACT system (BioMérieux, Marcy-l’Etoile, France) was used for strain identification as well as the antimicrobial susceptibility testing. The results of antimicrobial susceptibility testing were interpreted according to the Clinical and Laboratory Standards Institute (CLSI M100–S32) (CLSI, 2022). Species identification of the strain AP8900 was also identified by 16S rRNA gene sequencing [24].

### 2.2. Whole-Genome Sequencing, Assembly, and Annotation

Whole-genome sequencing of the strain AP8900 was conducted using both Illumina NovaSeq 6000 platform and PacBio Sequel IIe platform. The assembly of PacBio reads was performed using Hifiasm (version 0.13-r308)/Canu (version 1.7), and then assembly polishing were conducted using Pilon (version 1.22) [25] with Illumina reads. The completed genome of strain AP8900 were submitted to the NCBI GenBank database [26] and annotated using the NCBI Prokaryotic Annotation Pipeline [27].

### 2.3. Bioinformatics Analysis Towards the Genome of Strain AP8900

Acquired antibiotic resistance genes (ARGs) carried by the genome of strain AP8900 were detected using software ResFinder version 4.1 [28] at 90% identity and 60% coverage (default parameters of ResFinder). Insertion sequences (ISs) adjacent to ARGs in the genomes of the strain AP8900 were identified by submitting the corresponding gene sequences to ISfinder [29] with default parameters. Nucleotide sequence similarity analysis was performed using MegaBLAST (https://blast.ncbi.nlm.nih.gov/Blast.cgi?PROGRAM=blastn&PAGE_TYPE=BlastSearch&LINK_LOC=blas%E2%80%A6, accessed on 19 August 2025) (optimized for aligning DNA queries) [30] against the nucleotide non-redundant (nr) database of GenBank. The sequence comparison was performed and visualized using Easyfig version 2.2.5 [31] and BRIG version 0.95 [32].

### 2.4. Identification of the Bacteria Harboring Chromosome-Borne bla_NDM_ Available in GenBank Database

We performed MegaBLAST analysis of the *bla*_NDM-1_ gene sequence against the GenBank non-redundant (nr) database to identify sequences harboring *bla*_NDM_, applying thresholds of 100.00% coverage and >99.00% identity. Then, we selected the bacterial strains harboring chromosome-borne *bla*_NDM_ with complete genome. The variants of *bla*_NDM_ were further determined by software ResFinder 4.1 [28], and the genetic contexts of the chromosome-borne *bla*_NDM_ genes were analyzed using software ISfinder [29].

## 3. Results

### 3.1. Antibiotic Resistance Profiles of the Carbapenem-Resistant and Multidrug-Resistant A. pittii Strain AP8900

Antimicrobial susceptibility testing results indicated that the *A. pittii* strain AP8900 in our study exhibited resistance to the cephalosporins (cefepime and ceftazidime), carbapenem (imipenem), quinolones (ciprofloxacin and levofloxacin), sulfamethoxazole/trimethoprim, and β-lactam/β-lactamase inhibitor combinations (ticarcillin/clavulanate and piperacillin/tazobactam) (Table 1). Additionally, it also exhibited an intermediate resistance to meropenem (Table 1).

### 3.2. Genomic Characteristics of the Carbapenem-Resistant A. pittii Strain AP8900

The fully sequenced genome of the *A. pittii* strain AP8900 compised a circular chromosome with 3,966,182 bp (GenBank accession CP123765) and four plasmids, including one 86,394 bp plasmid (pAP8900-1, GenBank accession CP123766), one 43,600 bp plasmid (pAP8900-2, GenBank accession CP123767), one 12,558 bp plasmid (pAP8900-3, GenBank accession CP123768), and one 11,346 bp plasmid (pAP8900-4, GenBank accession CP123769).

The results of ResFinder showed that the acquired ARGs were distributed on both chromosome and the plasmids (pAP8900-1 and pAP8900-2). The chromosome of *A. pittii* strain AP8900 carried three beta-lactam resistance genes (*bla*_ADC-25_, *bla*_NDM-1_, and *bla*_OXA-526_). The plasmid pAP8900-1 carried one sulphonamide resistance gene (*sul*2). The plasmid pAP8900-2 harbored the acquired ARGs including genes encoding resistance to macrolide (*msr*(*E*) and *mph*(*E*)), tetracycline (*tet*(*39*)- *tetR*), chloramphenicol (*cml*), sulphonamide (*sul*2), and aminoglycoside (*aph*(*3″*)*-Ib* and *aph*(*6*)*-Id*).

Genetic context of chromosome-borne *bla*_NDM-1_ in the carbapenem-resistant *A. pittii* strain AP8900

**Figure 1:**
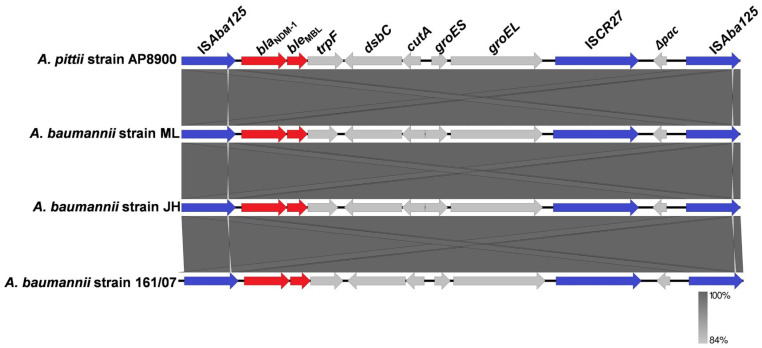
Comparison of the composite transposon Tn*125* (harboring the chromosome-borne *bla*_NDM-1_) carried by AP8900 with those of *A. baumannii* isolate ML, *A. baumannii* isolate JH, and *A. baumannii* 161/07, which were the initially identified Tn*125*. Resistance genes, ISs, and other genes are shown in red, blue, and gray, respectively.

The chromosome-borne *bla*_NDM-1_ of AP8900 was located on the approximately 10-kb composite transposon Tn*125*, bracketed by two copies of insertion sequence IS*Aba125* in the same orientation. The *bla*_NDM-1_ was flanked by IS*Aba125* and a bleomycin resistance gene, *ble*_MBL_, located adjacent to *trpF*-*dsbC*-*cutA*-*groES*-*groEL*. The IS*CR27* was found to be located downstream of the *groEL.* The Tn*125* of *A. pittii* AP8900 was nearly identical to those of *A. baumannii* 161/07 (100.00% coverage and 99.96% identity), *A. baumannii* isolate JH (100.00% coverage and 99.87% identity), and *A. baumannii* isolate ML (100.00% coverage and 99.86% identity), which were initially identified Tn*125* (Figure 1).

### 3.3. Bacteria Harboring Chromosome-Borne bla_NDM_ Available in GenBank Database

**Figure 2:**
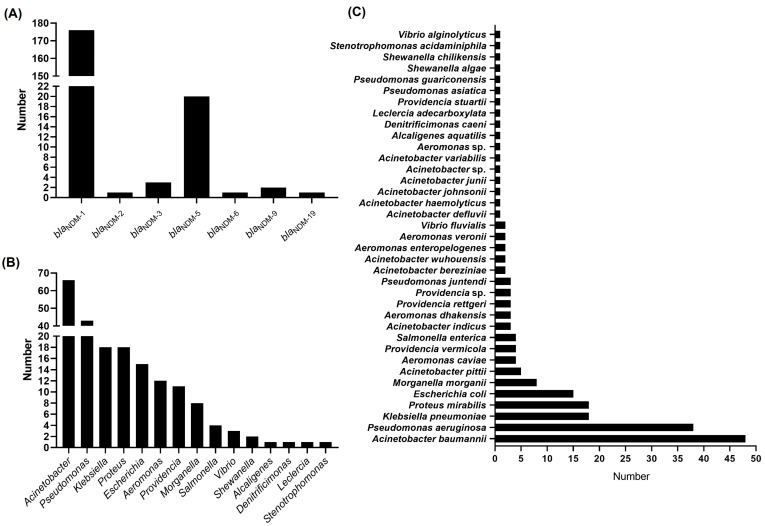
Statistics of bacterial hosts harboring chromosome-borne *bla*_NDM_. (**A**) Histogram of a number of variants of *bla*_NDM_ genes among the 204 bacterial strains with complete genomes harboring the chromosome-borne *bla*_NDM_. (**B**) Histogram about the number of the chromosome-borne *bla*_NDM_ distributed in different genera. (**C**) Histogram about the number of the chromosome-borne *bla*_NDM_ distributed in different species.

**Figure 3:**
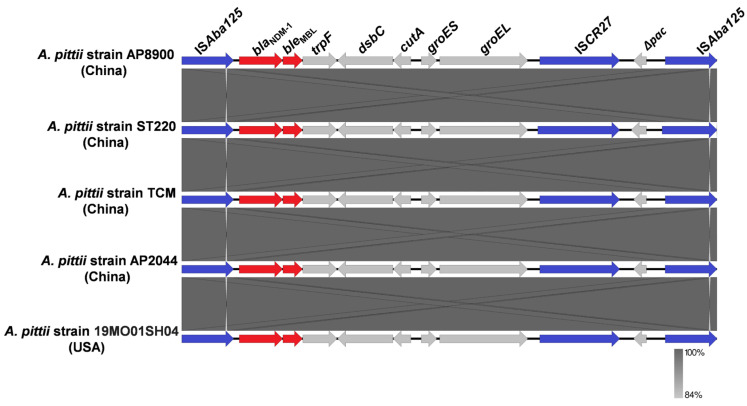
Comparison of the composite transposon Tn*125* carried by the five strains of *A. pittii* with complete genomes harboring chromosome-borne *bla*_NDM-1_. Resistance genes, ISs, and other genes are shown in red, blue, and gray, respectively.

BLASTn analysis using the DNA sequence of the *bla*_NDM-1_ gene of AP8900 against the GenBank nr database (24 December 2024) revealed 2774 total hits, of which 204 were complete genome sequences harboring the chromosome-borne *bla*_NDM,_ with *bla*_NDM-1_ being the most dominant (176 strains harboring *bla*_NDM-1_), followed by the *bla*_NDM-5_ (20 strains harboring *bla*_NDM-5_) (Figure 2A). The hosts harboring chromosome-borne *bla*_NDM_ were widespread, involving 15 different genera, with *Acinetobacter* (66 strains), *Pseudomonas* (43 strains), *Klebsiella* (18 strains), *Proteus* (18 strains), and *Escherichia* (15 strains) being the TOP5 genera (Figure 2B). At the species level, the most common species harboring chromosome-borne *bla*_NDM_ was *A. baumannii* (48 strains), followed by *Pseudomonas aeruginosa* (38 strains), *K. pneumoniae* (18 strains), *Proteus mirabilis* (18 strains), and *Escherichia coli* (15 strains) (Figure 2C). So far, only five strains of *A. pittii* with complete genomes harboring chromosome-borne *bla*_NDM_ (all *bla*_NDM-1_) were found, including four strains from China (Hefei, Hangzhou, Luzhou, and Zhuhai) and one strain from the USA (Figure 3). Notably, four of these five strains harbored the nearly identical Tn*125* carried by AP8900 in our study (100.00% coverage and >99.00% identity) (Figure 3). The *A. pittii* strain AP8900 was the first reported with a complete genome in southern China.

The distribution of Tn*125* carried by AP8900 in this study was also explored in other strains with complete genomes harboring chromosome-borne *bla*_NDM_. Based on the DNA sequence of Tn*125* carried by AP8900, the BLAST searches against the 204 bacterial strains with complete genome harboring chromosome-borne *bla*_NDM_ indicated that the Tn*125* carried by AP8900 was present on 57 bacterial strains (100.00% coverage and >99.00% identity). Notably, 49 of the 57 bacterial strains harboring the 10-kb Tn*125* carried by AP8900 were found to belong to the *Acinetobacter* genus, including 11 species, accounting for 85.96% of all the 57 bacterial strains carrying Tn*125* carried by AP8900. In addition, Tn*125* carried by AP8900 was also found in *P. mirabilis* (four strains), *K. pneumoniae* (three strains), and *Morganella morganii* (one strain) (Table 2).

### 3.4. Genomic Analysis of the Antibiotic-Resistant Plasmids Carried by A. pittii Strain AP8900

**Figure 4:**
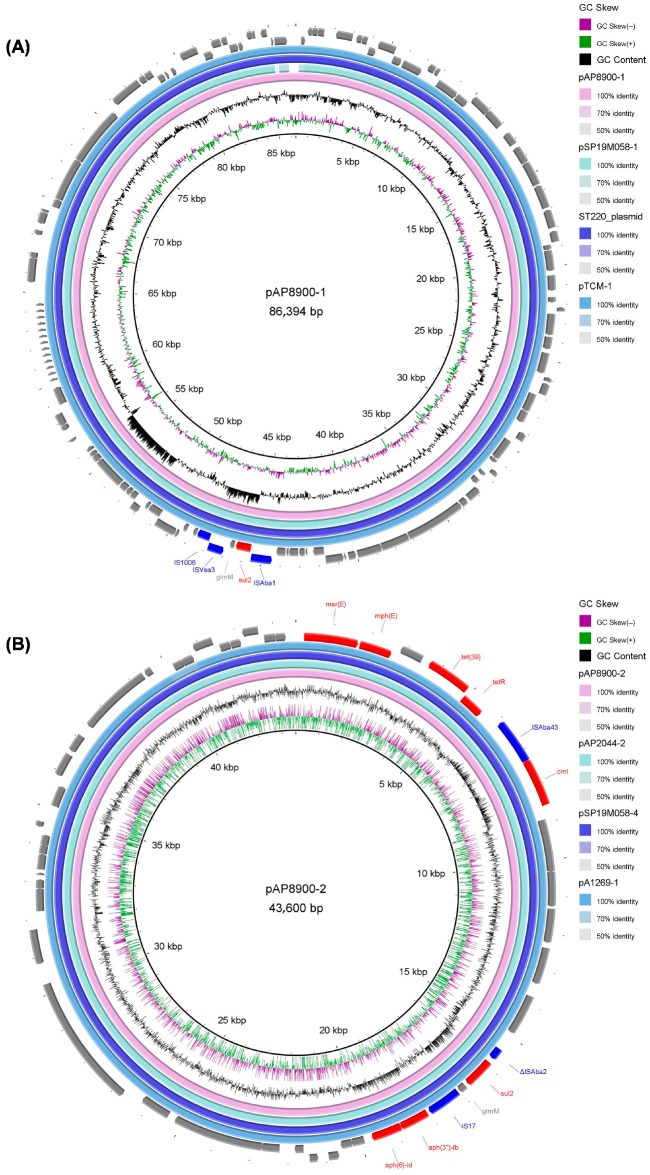
Genetic structures of the two antibiotic resistance plasmids were found in *A. pittii* strain AP8900. (**A**) Comparison of plasmid pAP8900-1 and the plasmids was highly similar to pAP8900-1. (**B**) Comparison of plasmid pAP8900-2 and the plasmids was highly similar to pAP8900-2. Resistance and transposase are shown in red and blue, respectively.

For the plasmid pAP8900-1 of *A. pittii* strain AP8900, the sulphonamide resistance gene (*sul*2) and one phosphoglucosamine mutase gene (*glmM*) were bracketed by *ISAba1* and IS*Vsa3*-IS*1006*. For the pAP8900-2, one insertion sequence IS*Aba43* was located upstream of the chloramphenicol resistance gene *cml*, adjacent to the macrolide resistance genes *msr*(*E*)-*mph*(*E*) and the tetracycline resistance genes *tet*(*39*)-*tetR*. Notably, the “*sul2*-*glmM*” present in the pAP8900-1 was also found on the pAP8900-2, which was bracketed by ∆IS*Aba2* and IS*17*, adjacent to the aminoglycoside resistance genes *aph*(*3″*)*-Ib*-*aph*(*6*)*-Id* (Figure 4).

Based on the results of the BLAST search against the GenBank nr database, the 86.39-kb plasmid pAP8900-1 had high similarity with the plasmid pSP19M058-1 in *A. pittii* strain 19MO01SH04 (99.00% coverage and 99.99% identity), the plasmid in *A. pittii* strain ST220 (100.00% coverage and 99.82% identity), and the plasmid pTCM-1 in *A. pittii* strain TCM (100.00% coverage and 99.93% identity) (Figure 4A). In addition, BLAST search against the GenBank nr database also showed that the 43.60-kb plasmid pAP8900-2 was highly similar to the plasmid pAP2044-2 in *A. pittii* strain AP2044 (100.00% coverage and 99.92% identity), the plasmid pSP19M058-4 in *A. pittii* strain 19MO01SH04 (100.00% coverage and 99.99% identity), and the plasmid pA1269-1 in *A. pittii* strain A1269 (100.00% coverage and 100.00% identity) (Figure 4B).

## 4. Discussion

In this study, the carbapenem-resistant *A. pittii* strain AP8900 harbored the chromosome-borne carbapenemase gene *bla*_NDM-1_. The NDM-type carbapenemases are quickly spreading and the troublesome family of Ambler class B β-lactamases, and NDM-producing *Acinetobacter* spp., has been recently reported in numerous countries, particularly the predominant NDM-1 type [7,8]. The first global report of NDM-producing clinical *Acinetobacter* strain was made in 2010 when *bla*_NDM-1_-positive *A. baumannii* was found in India [33]. The first report of blaNDM-1-positive clinical isolates in China occurred in 2010, with four *A. baumannii* isolates identified in different provinces [34]. Since then, *bla*_NDM-1_ has been detected numerous times in different strains of *Acinetobacter*, such as *Acinetobacter junii*, *Acinetobacter lwoffii*, and *A. pittii*, which were isolated from the clinical, environmental, and farm animal samples in China [34,35,36,37]. In this study, we found that the bacterial hosts harboring chromosome-borne *bla*_NDM_ were widespread, involving 15 different genera, with *Acinetobacter* and *Pseudomonas* being the most common. Infections caused by carbapenem-resistant Gram-negative bacteria (GNB) pose a significant threat due to their high morbidity and mortality rates [38,39]. Infections caused by NDM-producing bacteria are generally more challenging to treat than those caused by KPC-producing bacteria. For example, ceftazidime–avibactam (CAZ-AVI), a novel last-resort β-lactam antibiotic, is effective against KPC-producing bacterial infections but lacks activity against NDM-producing strains [40]. Notably, new antibiotics classes such as Trojan Horse antibiotics [41] and antibiotics targeting bacterial metallophores [42] represent promising therapeutic options for infections caused by NDM-producing strains.

In this study, the *bla*_NDM-1_ of strain AP8900 was found to locate on the composite transposon Tn*125*, which is bracketed by two IS*Aba125*s in the same orientation, forming the structure “IS*Aba125-bla*_NDM-1_-*ble*_MBL_-*trpF*-*dsbC*-*cutA*-*groES*-*groEL*-IS*CR27*-IS*Aba125*.” Notably, only five strains of *A. pittii* with complete genomes harboring chromosome-borne *bla*_NDM_ (all *bla*_NDM-1_) were found, all carrying the nearly identical Tn*125* carried by AP8900, which was highly similar to the initially identified Tn*125* [22]. The 1087 bp insertion sequence IS*Aba125* carried by Tn*125* belongs to a member of the IS30 family of insertion elements [22,43]. It has been suggested that the *bla*_NDM_ gene has been acquired into *Acinetobacter* spp. from the environment and then has spread to the *Enterobacteriaceae* with the help of the IS*Aba125* [44]. Notably, for the Tn*125*, the IS*Aba125* upstream of the *bla*_NDM_ has been reported to provide the -35 region of a promoter necessary for the expression of *bla*_NDM-1_ [45]. In addition to *bla*_NDM-1_, the IS*Aba125* was also reported to activate the horizontal transfer of *ampC* gene among *A. baumannii* strains leading to cephalosporin resistance [46]. The *groES-groEL-*IS*CR27* section (adjacent to the other copy of IS*Aba125*) may originate from *Xanthomonas* sp. [47]. It is hypothesized that IS*CR27*, employing a rolling-circle transposition mechanism [48], initially mediated the ancestral mobilization of *bla*_NDM_ in *Xanthomonas* sp. and precisely positioned it downstream of IS*Aba125* [22,47]. Moreover, the consistent presence of IS*Aba125* in all *bla*_NDM_-positive strains so far, along with early observations in *A. baumannii*, proposed that Tn*125* was the ancestral transposon facilitating the mobilization of *bla*_NDM_, and *A. baumannii* was its ancestral host [47,49].

In this study, two antibiotic resistance plasmids were found in *A. pittii* strain AP8900. Notably, the configuration “*sul2*-*glmM*” was found to locate both on the pAP8900-1 (*ISAba1*-*sul2*-*glmM*-IS*Vsa3*-IS*1006*) and the pAP8900-2 (∆IS*Aba2*-*sul2*-*glmM*-IS*17*) of the *A. pittii* strain AP8900. The “*sul2*-*glmM*” was also reported in other configurations of *Acinetobacter* species. For example, the configuration “IS*Aba1*-*sul2*-*glmM*-∆IS*CR2*”, which was part of Tn*6450*, was found in *Acinetobacter towneri* strain SWBY1 [50]; the configuration “IS*Aba*1-*sul2*-∆*glmM*-IS*CR2*-*strB*-*strA*” was detected in *A. baumannii* RUH 875 [51]; and the configuration “IS*Aba1*-*sul2*-*glmM*-IS*Vsa3*-*lysR*-*floR*-DUF3363-*strB*-*strA*-IS*Aba1*” was identified in *Acinetobacter johnsonii* Acsw19 [52].

## 5. Conclusions

In summary, we report the complete genome of *A. pittii* strain AP8900 harboring chromosome-borne *bla*_NDM-1_, which was isolated from the sputum of a 60-year-old patient. In strain AP8900, the chromosome-borne *bla*_NDM-1_ was located on the Tn*125* composite transposon, which was bracketed by two copies of IS*Aba125* in the same orientation. In addition, two antibiotic resistance plasmids were identified in *A. pittii* strain AP8900. The complete genome of *A. pittii* AP8900 strain from southern China provides important data for the analysis of antimicrobial resistance in this region.

## Figures and Tables

**Table 1:** Minimum inhibitory concentration (MIC) values of the *A. pittii* strain AP8900.

Antibiotics		MIC (μg/mL)	Interpretation
Categories	Name		
Cephalosporins	Ceftazidime	≥64	R
	Cefepime	≥32	R
Carbapenems	Imipenem	8	R
	Meropenem	4	I
Fluoroquinolones	Ciprofloxacin	≥4	R
	Levofloxacin	≥8	R
Sulfonamides	Sulfamethoxazole/trimethoprim	160	R
β-lactam/β-lactamase inhibitor combinations	Piperacillin/tazobactam	≥128	R
	Ticarcillin/clavulanate	≤8	R

Note: R, Resistant; I, Intermediate.

**Table 2:** The Tn*125* carried by strain AP8900 distributed in the bacterial strains with complete genome harboring chromosome-borne *bla*_NDM_.

Species	Number of Strains Harboring Tn*125*	Number of Strains Harboring Chromosome-Borne *bla*_NDM_	Proportion * (%)
*Acinetobacter baumannii*	31	48	64.58
*Acinetobacter bereziniae*	2	2	100.00
*Acinetobacter defluvii*	1	1	100.00
*Acinetobacter haemolyticus*	1	1	100.00
*Acinetobacter indicus*	3	3	100.00
*Acinetobacter johnsonii*	1	1	100.00
*Acinetobacter junii*	1	1	100.00
*Acinetobacter pittii*	5	5	100.00
*Acinetobacter* sp.	1	1	100.00
*Acinetobacter variabilis*	1	1	100.00
*Acinetobacter wuhouensis*	2	2	100.00
*Klebsiella pneumoniae*	3	18	16.67
*Morganella morganii*	1	8	12.50
*Proteus mirabilis*	4	18	22.22

Note: * Proportion represents the percentage of strains harboring Tn*125* carried by AP8900 among those carrying chromosome-borne *bla*_NDM_.

## Data Availability

The complete genome sequences of the strain AP8900 in this study have been submitted to the GenBank nucleotide database with accession numbers CP123765-CP123769.

## Abbreviations

The following abbreviations are used in this manuscript:

NDM	New Delhi metallo-beta-lactamase
ARGs	Directory of open access journals antibiotic resistance genes
